# Predicting outcomes after blunt chest wall trauma: development and external validation of a new prognostic model

**DOI:** 10.1186/cc13873

**Published:** 2014-05-14

**Authors:** Ceri Elisabeth Battle, Hayley Hutchings, Simon Lovett, Omar Bouamra, Sally Jones, Aruni Sen, James Gagg, David Robinson, Jake Hartford-Beynon, Jeremy Williams, Adrian Evans

**Affiliations:** 1 College of Medicine, Swansea University, Swansea, Wales, UK; 2 Physiotherapy department, Morriston Hospital, Swansea, Wales, UK; 3 NISCHR Haemostasis Biomedical Research Unit, Swansea, Wales, UK; 4 Trauma Audit Research Network, University of Manchester, Salford, UK; 5 Royal Gwent Hospital, Newport, Wales, UK; 6 Wrexham Maelor Hospital, Wrexham, Wales, UK; 7 Musgrove Park Hospital, Taunton, UK; 8 Bradford Royal Infirmary, Bradford, UK; 9 Ysbyty Gwynedd Hospital, Bangor, Wales, UK; 10 Glangwili General Hospital, Carmarthen, Wales, UK

## Abstract

**Introduction:**

Blunt chest wall trauma accounts for over 15% of all trauma admissions to Emergency Departments worldwide. Reported mortality rates vary between 4 and 60%. Management of this patient group is challenging as a result of the delayed on-set of complications. The aim of this study was to develop and validate a prognostic model that can be used to assist in the management of blunt chest wall trauma.

**Methods:**

There were two distinct phases to the overall study; the development and the validation phases. In the first study phase, the prognostic model was developed through the retrospective analysis of all blunt chest wall trauma patients (*n* = 274) presenting to the Emergency Department of a regional trauma centre in Wales (2009 to 2011). Multivariable logistic regression was used to develop the model and identify the significant predictors for the development of complications. The model’s accuracy and predictive capabilities were assessed. In the second study phase, external validation of the model was completed in a multi-centre prospective study (*n* = 237) in 2012. The model’s accuracy and predictive capabilities were re-assessed for the validation sample. A risk score was developed for use in the clinical setting.

**Results:**

Significant predictors of the development of complications were age, number of rib fractures, chronic lung disease, use of pre-injury anticoagulants and oxygen saturation levels. The final model demonstrated an excellent c-index of 0.96 (95% confidence intervals: 0.93 to 0.98).

**Conclusions:**

In our two phase study, we have developed and validated a prognostic model that can be used to assist in the management of blunt chest wall trauma patients. The final risk score provides the clinician with the probability of the development of complications for each individual patient.

## Introduction

Blunt chest-wall trauma accounted for over 15% of all trauma admissions to Emergency departments (EDs) worldwide [1]. Reported mortality ranges between 4 and 60%, however, no current national guidelines exist to assist in the management of this patient group unless the patient has severe, immediate life-threatening injuries [2]. The difficulties in the management of the blunt chest-wall trauma patient are becoming increasingly well recognised in the literature [3,4]. The blunt chest-wall trauma patient commonly presents to the ED initially with no respiratory difficulties, but can develop respiratory complications approximately 48 to 72 hours later [5,6]. Clinical symptoms are not considered an accurate predictor of outcome following non-life threatening blunt chest-wall trauma [7]. Decisions on the appropriate level of care required by the patient following discharge from the ED are therefore difficult, which is further compounded by the lack of current national guidelines. A number of well-documented risk factors for morbidity and mortality exist for blunt chest-wall trauma, including patient age, pre-existing disease, number of ribs fractured and the onset of pneumonia during the recovery phase [2,8].

A prognostic model enables the clinician to use combinations of predictor values to estimate a probability that a specified outcome will occur [9]. The resulting model may be used to divide the patient into categories of risk or predict probabilities of a pre-specified outcome [9]. A number of models exist for blunt chest trauma, however, most are designed for use with patients with multiple injuries and very few have been externally validated or presented in a clinically practical way [3]. For the purpose of this study, blunt chest-wall trauma was defined as blunt chest injury resulting in chest wall contusion or rib fractures, with or without non-immediate life-threatening lung injury [2]. We have developed and validated a prognostic model for the development of complications following blunt chest-wall trauma. Using the results of the prognostic model, we have also developed a simple risk score for use in the clinical setting which can assist the clinician in the management of the blunt chest-wall trauma patient.

## Materials and methods

There were two distinct phases to the overall study; the development and the validation phases. Published guidelines for prognostic model development were followed throughout the completion of this work [9-12]. These guidelines outlined the stages of model development and the appropriate statistical analysis that should be undertaken at each phase [9-12].

### Development phase study design

Data were collected retrospectively from the medical notes of each patient. If there was no record in the patient’s notes of chronic lung disease, cardiovascular disease, use of pre-injury anticoagulants or current smoking status, then it was assumed that these predictors were absent. The number of rib fractures was determined from the chest radiograph if not documented in the medical notes.

### Patients

The prognostic model was developed on a sample of 276 patients who presented to the ED of a large regional trauma centre in South Wales between 2009 and 2011, with a primary diagnosis of blunt chest-wall trauma. Patients were excluded if they were under 18 years of age or if they had sustained any immediate life-threatening injury. As eight prognostic variables were under investigation, a total of 80 blunt chest-wall trauma patients who developed complications were needed in the analysis [13].

### Outcomes

The development of complications following blunt chest-wall trauma was the composite outcome measure investigated in this study. Data collection for this outcome was completed from the time the patient presented to the ED through to discharge from hospital. Patients were reported to have developed complications if one or more of the following were documented in their medical records; in-hospital mortality, morbidity including all pulmonary complications (chest infection, pneumonia, haemothorax, pneumothorax, pleural effusion, or empyema), ICU admission, or a prolonged length of stay as defined as a total hospital stay of seven or more days [14,15].

### Prognostic variables

For the development of the prognostic model, we considered age, number of rib fractures, chronic lung disease, cardiovascular disease, use of pre-injury anti-coagulants, smoking status and oxygen saturations and respiratory rate on initial assessment in the ED. These risk factors were based on previous research [2,8,16].

### Analysis

Baseline characteristics were presented as median and interquartile range (due to non-normal distributions) for the continuous variables and numbers and percentages for categorical variables. Differences between the baseline characteristics in the development and validation samples were analysed using the Mann-Whitney *U*-test (continuous variables) and Fisher’s exact test (categorical variable). Odds ratios and 95% CI were presented from the univariable analysis. All significant prognostic variables at 5% significance on the univariable analysis were included in final analysis. Multivariable logistic regression analysis using fractional polynomials (to assess linearity of the continuous variables) identified five significant predictors using the Akaike information criterion with a backward elimination approach. We quantified the predictive contribution of each variable by its *z*-score (the regression coefficient divided by its standard error). There was less than 2% missing data, therefore we used a simple imputation method to avoid exclusion of patients from the final analysis [17].

### Performance of the model

The final prognostic model was based on five prognostic variables which were significant at the *P* <0.05 level following multivariable logistic regression analysis. We assessed model performance using analysis of calibration and discrimination. Calibration was assessed graphically and with the Hosmer-Lemeshow test. Discrimination was assessed with the *c*-statistic (equivalent to the area under the receiver operator curve) [17].

### Validation phase study design

A multi-centre prospective study design was used in order to externally validate the model, in which seven hospitals in England and Wales between 2012 and 2013 participated in data collection. A total of 237 patients was included, which was a sufficient sample size for a validation study [18]. The model was validated by comparing predicted versus observed outcomes and performance assessed using calibration and discrimination analysis. As a result of the lower calibration in the validation model, the final model was updated through the application of a correction factor to adjust the slope intercept of the original model. This technique is outlined in the guidelines by Janssen *et al*. [19]. Sensitivity, specificity, positive and negative predictive values were calculated for the final model.

### Score development

We developed a simple clinical score based on the regression coefficients from the final model. To calculate the risk score for the individual predictors, the coefficient of each predictor was multiplied by a factor so that the smallest coefficient was transformed into a value close to one. This method was adapted from previous studies [20,21]. These individual scores were then added together to provide an overall risk score for each patient. Using the validation sample, each patient’s final overall risk score was compared to their probability of developing complications initially calculated using the final logistic regression equation. The individual final risk scores were categorised into groups (0 to 10, 11 to 15, 16 to 20, 21 to 25, 26 to 30 and ≥30) and the mean and standard deviation of all the corresponding probabilities were calculated. This would provide the clinician with a probability of the development of complications for each possible final risk score. Estimates of appropriate cut off points were included guiding the clinician as to whether the patient could be safely discharged home, or whether they should be admitted to a ward or intensive care unit.

### Ethical approval

Full ethical approval for this study was granted by the South West Wales Research Ethics Committee. Written consent to participate and publish study findings was provided by each recruited patient.

## Results

### General characteristics

Table 1 illustrates the baseline characteristics of the patients comparing the development and validation samples. Significant differences were recorded in rate of outcome and most predictors between the two groups. More of the patients were male (64%) and the most common injury mechanisms were fall (72%), road traffic accident (14%), sporting injury (9%) and assault (3%). A total of 31 (6%) deaths was recorded in both samples.

**Table 1:** Comparison between baseline characteristics of patients in the development and validation samples

	**Development sample total**	**No complications**	**Complications**	**Validation sample total**	**No complications**	**Complications**	***P*-value**
	274	113 (41%)	161 (59%)	237	161 (57%)	103 (43%)	<0001*
**Age**	69 (28)	62 (30)	76 (21)	57 (34)	47 (31)	71 (29)	<0001*
**Number of rib fractures**	3 (3)	2 (3)	3 (1)	1 (3)	0 (1)	3 (3)	<0.001*
**Oxygen saturations**	95 (5)	96 (4)	94 (6)	97 (5)	98 (2)	95 (7)	<0.001*
**Respiratory rate**	18 (6)	18 (4.5)	20 (6.5)	18 (6)	16 (2)	20 (6)	0.062
**Chronic lung disease**	154 (56%)	38 (34%)	116 (72%)	49 (21%)	13 (8%)	36 (35%)	<0.001*
**Cardiovascular disease**	116 (42%)	34 (30%)	82 (51%)	53 (22%)	13 (8%)	40 (39%)	<0.001*
**Smoker**	92 (34%)	43 (38%)	49 (30%)	67 (28%)	40 (25%)	27 (26%)	0.213
**Pre-injury anticoagulants**	117 (43%)	28 (25%)	89 (55%)	47 (20%)	6 (4%)	41 (40%)	<0.001*

Number (percentages), median (interquartile range). *Significant difference (*P*-value) between totals in development and validation samples.

### Development phase univariable analysis

Results of the univariable analysis (in the development sample only) highlighted a number of significant predictors for the development of complications. Unadjusted odds ratios and the 95% CI are included for each of the categorical variables.

### Development phase multivariable analysis

All eight predictors from Table 2 were included in the analysis (from the development sample only). Five significant predictors were included in the final model: age, number of rib fractures, chronic lung disease, use of pre-injury anticoagulants and oxygen saturations (Table 3). Continuous variables were analysed as linear terms as there was no indication of non-linearity when analysed using the multivariable fractional polynomials. The number of rib fractures and chronic lung disease were the strongest predictors of the development of complications following blunt chest-wall trauma.

**Table 2:** Univariable analysis: unadjusted odds ratios for the predictors of the development of complications

	**No complications n = 113**	**Complications n = 161**	***P*-value**	**Unadjusted odds ratios (95% CI)**
Age	47 31	71 (30.0)	0.0001*	
Number of rib fractures	0 (1.0)	3 (1.0)	0.0001*	
Oxygen saturations	98 (2.0)	95 (7.0)	0.0001*	
Respiratory rate	18 (6.0)	20 (8.0)	0.0794	
Chronic lung disease	38 (34%)	116 (72%)	0.0001*	5.1 (3.0, 8.6)
Pre-injury anticoagulants	28 (25%)	89 (55%)	0.0001*	3.8 (2.2, 6.4)
Cardiovascular disease	34 (30%)	82 (51%)	0.0008*	2.4 (1.5, 4.0)
Smoking status	43 (38%)	49 (30%)	0.1964	0.7 (0.4, 1.2)

Number and percentages, median (interquartile). *Significant difference (*P*-value).

**Table 3:** Multivariable predictive model

**Predictor**	**Odds ratio (95% CI)**	***z*-score**
Age^a^	1.0 (1.0, 1.0)	1.80
Number of rib fractures^b^	1.5 (1.3, 1.9)	4.21
Chronic lung disease	2.2 (1.2, 4.1)	2.50
Pre-injury anticoagulants	1.9 (1.0, 3.7)	1.91
Oxygen saturations^c^	0.9 (0.9, 1.0)	−1.55

^a^Per one year increase; ^b^per one fracture increase; ^c^per 1% decrease of oxygen saturations.

### Performance of model

**Figure 1:**
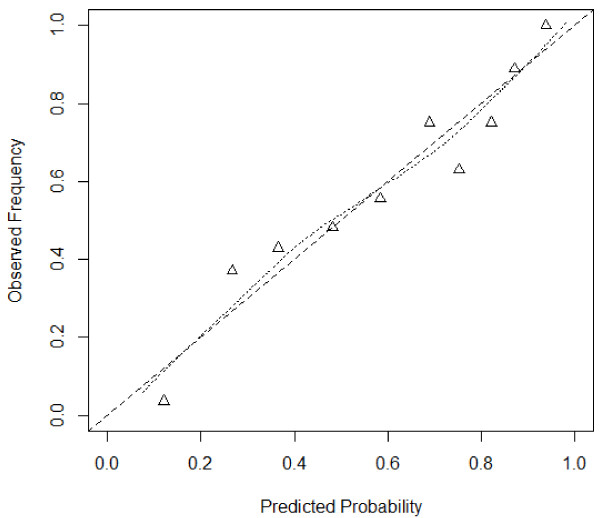
**Calibration of model using expected and observed probabilities of development of complications.** Triangles show risk of outcome in tenths of patients with similar predicted probabilities. Dotted line: relationship between observed frequency and predicted probability of development of complications; broken line: ideal relationship between observed and predicted frequency of outcome in model with perfect calibration.

The model showed excellent discrimination with a *c*-statistic of 0.80 (95% CI 0.75, 0.85). The model calibration is illustrated in the observed versus predicted outcomes graph in Figure 1. The model showed good calibration when evaluated with the Hosmer Lemeshow test (9.22, *P* = 0.32).

### External validation

**Figure 2:**
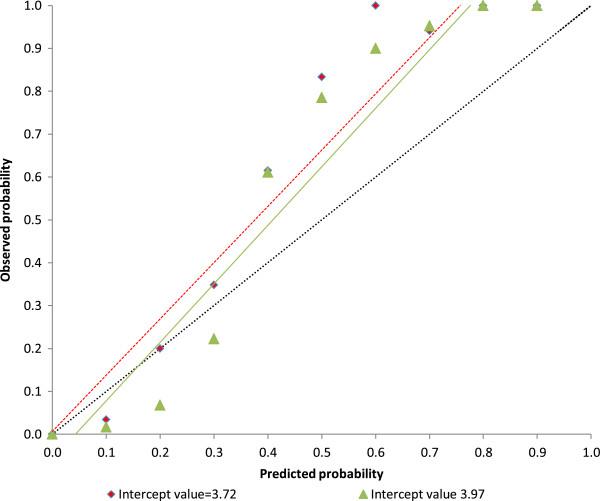
**External validation model calibration using observed versus predicted outcomes.** Red diamonds show risk of outcome in tenths of patients with similar predicted probabilities with intercept value of 3.72. Green triangles show risk of outcome in tenths of patients with similar predicted probabilities with updated intercept value of 3.97. Black broken line: ideal relationship between observed and predicted frequency of outcome in model with perfect calibration; red broken line: relationship between observed frequency and predicted probability of development of complications in original validation model; green line: relationship between observed frequency and predicted probability of development of complications in updated validation model.

In the validation phase of the study, the model was prospectively externally validated in seven hospitals in England and Wales. Discrimination of the final validation model was excellent with a *c*-index of 0.96 (95% CI 0.93, 0.98), although an increase in the *c*-statistic is unusual in a validation study. As expected, calibration was poorer in the validation model (Figure 2), therefore, we updated the model. The slope intercept of the development model was adjusted using a correction factor to correspond with the lower complication rate in the validation sample, which gave an improved discrimination and calibration (Figure 2). The results demonstrated that final model sensitivity was 80%, specificity was 96%, positive predictive value was 93% and negative predictive value was 86%.

### Clinical score

Based on the results of the logistic regression analysis, we developed a simplified clinical score by transforming the regression coefficient of each predictor into an equivalent accurately weighted risk integer score (Table 4). For example, a 67-year-old (score 6) patient with three rib fractures (score 9), a history of chronic lung disease (score 5) and oxygen saturations of 87% (score 4) would have a final risk score of 24 points.

**Table 4:** Risk factor scores as transformed from the regression coefficients

	**Regression coefficient**	**Risk score**
Age	0.0162	1^a^
Number of rib fractures	0.418	3^b^
Chronic lung disease	0.789	5
Pre-injury anticoagulant use	0.637	4
Oxygen saturation levels	−0.0651	2^c^

^a^Per additional 10-year increase starting at 10 years of age; ^b^per additional rib fracture; ^c^per 5% decrease in oxygen saturations starting at 94%.

Table 5 illustrates the final risk scores and their corresponding probability of developing complications following blunt chest-wall trauma. Using these results, for example, it is possible to estimate that a patient who scores 12 has a 29% (±8) probability of developing complications compared to a patient with a final risk score of 36 who has an 88% (±7) probability of developing complications.

**Table 5:** Final risk scores and corresponding probability of developing complications

**Final risk score**	**Probability mean ± SD**
0 to 10	13% ± 6
11 to 15	29% ± 8
16 to 20	52% ± 8
21 to 25	70% ± 6
26 to 30	80% ± 6
31+	88% ± 7

A final total risk score of ≥11 was the most accurate cut off point (in terms of sensitivity, specificity and predictive values) at which the blunt chest-wall trauma patient was considered at risk of developing complications. The percentage of patients who would have been correctly managed (admitted to hospital and subsequently developed complications) was 90% and those correctly discharged directly from the ED (who had not developed complications) would have been 87%. If a final risk score ≥26 was selected as the cut off point at which the blunt chest-wall trauma patient was considered a high enough risk to require ICU admission, then the percentage of patients correctly managed (scored at ≥26 and the observed outcome was ICU admission) was 95%. The percentage of patients who would have been incorrectly managed (scored at ≥26 but were not admitted to the ICU in the observed outcome) would have been 11%.

## Discussion

We have developed and validated a prognostic model for predicting the development of complications following blunt chest-wall trauma. The final model has excellent discrimination suggesting that the clinician can confidently assess whether the patient with the higher risk prediction using the model will develop complications following blunt chest-wall trauma, compared to the patients with low risk predictions who will not develop complications. As expected, the validation model demonstrated poorer calibration and numerous authors have offered explanations for this result in a validation sample [22,23]. It is likely that the poor calibration was due to the significantly lower rates of the development of complications in the validation sample.

Common practice is simply to reject an original prediction model as a result of decreased predictive performance in the validation sample. A new prediction model is then developed and as a consequence the original dataset is neglected and clinicians are faced with numerous possible prediction models, very few of which have been externally validated for use in new samples [22]. Research now suggests that the model should be adjusted in order to improve its performance on the new population and this adjusted model is then based on both the original and validation data, further strengthening its stability and generalisability [9]. The model in this validation study was therefore updated using a previously described method known as recalibration [19,23]. By simply adjusting the intercept for the original model, the validation model calibration was improved.

Patient age, number of rib fractures, chronic lung disease, pre-injury anticoagulants and oxygen saturation levels were the significant risk factors for development of complications following blunt chest-wall trauma. Patient age, number of rib fractures and chronic lung disease have been reported as significant risk factors for poor outcomes in a number of recent studies and possible explanations for these factors have been previously discussed [2,16,24]. Pre-injury anticoagulant use and oxygen saturation levels have only been reported as risk factors for the development for complications following blunt chest-wall trauma in a previous study by Battle *et al*. [2] and therefore, further research into these risk factors would be beneficial [16].

The results of this study have demonstrated that risk can be easily and accurately stratified from simple demographic and clinical variables on initial assessment of the blunt chest-wall trauma patient in the ED. The risk factors are all currently routinely measured in the ED and do not require expensive, time-consuming or complicated technology to investigate. This is one of the most important factors in the success of prognostic model development according to previous research [9]. The clinician would simply collect routine data, total the scores for each risk factor, then obtain the corresponding probability of the development of complications. A more accurate decision can be made by the clinician on whether the patient is safe for discharge home directly from the ED, or whether the patient requires admission to hospital. Not only could this reduce the development of complications in blunt chest-wall trauma patients through close observation and early aggressive prophylactic treatment in the admitted patient, but also reduce unnecessary admission of patients unlikely to develop complications.

The overall results of this study suggest that the final validation model could be safely and effectively used in the clinical setting in England and Wales for assisting in the management of blunt chest-wall trauma patients. This is the first prognostic model that has been developed and externally validated in a prospective multi-centre study for use with blunt chest-wall trauma patients. The model can be used with the less severely injured patient who on presentation to the ED is not suffering any overt signs of respiratory distress, but will potentially go on to develop severe life-threatening pulmonary complications. Research has demonstrated that careful observation and early aggressive therapy can limit these complications, therefore identification of the high-risk patient is imperative for optimal management [25]. It is inevitable, however, that the final decision on patient management must be individualised and many factors that cannot be translated into a statistical model must be considered. The overall purpose of the prognostic model is simply to guide clinical decision-making, not replace it.

This study has a number of strengths and limitations. External validation using a prospective multi-centre trial is considered the most robust validation technique ensuring generalisability of the study’s results [9]. Current methodological recommendations for clinical prediction research, as outlined by Bouwmeester *et al*. [17] have been followed in the design and completion of the prognostic model for use with blunt chest-wall trauma patients. These recommendations included sample size and selection, clear definitions of risk factors and outcomes under investigation, handling of missing data, reporting of both univariable and multivariable results and calculation of model performance measures. The final model was also recalibrated as recommended by recent research [19]. As a result the reliability and applicability is sufficient that the model could be safely and effectively used in the clinical setting. The external validation results also confirm the clinical usefulness of the model in blunt chest-wall trauma management throughout England and Wales. It is important to emphasise, however, that the validation model *c*-statistic is a very unusual result and should be interpreted with caution. It is more common for the *c*-statistic to decrease in the validation study, rather than to increase as we found.

One of the limitations of this study is the loss of patients to follow up. Due to limited resources, it was not considered feasible to investigate the patients’ follow up once they had left hospital care. Any use of primary care for complications that developed following hospital discharge would not have been included in the study results. The data collection was not fully blinded as recommended by Bouwmeester *et al*. [17], however, the clinicians collecting the data in the validation study were blinded to which of the risk factors and outcomes were being used in the final analysis. Another limitation of the validation study concerns the timing of the data collection. For example, the patient’s oxygen saturation levels may have varied according to the time in which they were recorded. If the data were collected before analgesia was given in the ED, then the results may have been worse than if the patient had received analgesia and could breathe more easily. As a result of these limitations, the results of this study should be considered with caution.

There was a significantly lower rate of complications in the validation sample than the development sample. This could be explained by the protocol for management of the blunt chest-trauma patient in the different hospitals. For example, in the hospital where the original model was developed, patients are routinely admitted to the ICU if they need invasive analgesia such as an epidural, as this is where epidural patients are currently managed. In order to quantify the number of rib fractures sustained by the patient, a chest radiograph or computed tomography (CT) scan and its subjective interpretation is required. Due to the inherent difficulties in identification of rib fractures on chest radiographs, the clinician is advised to record the number of rib fractures identified on imaging, or suspected clinically following physical examination of the patient [26].

The use of a composite outcome measure could result in one element dominating the total outcome measure. This was not the case in this study, however, as apart from the low mortality rate, there was a relatively even number of the different types of reported complications.

## Conclusions

Blunt chest-wall trauma patients are often difficult to manage in the ED, due to the frequent onset of delayed complications. We have developed and validated a prognostic model, which can assist the clinician in the management of the blunt chest-wall trauma patient. The model has demonstrated excellent prediction capabilities and can be safely used in EDs managing this patient group.

## Key messages

• Blunt chest-wall trauma patients can be difficult to manage in the ED due to the frequent onset of delayed complications

• Risk factors for the development of complications following blunt chest-wall trauma were patient age, number of rib fractures, chronic lung disease, use of pre-injury anticoagulants and oxygen saturations

• The prognostic model developed and externally validated in this study can be safely used to assist in the management of the blunt chest-wall trauma patient in the ED

• The prognostic model demonstrates excellent predictive capabilities

## Abbreviations

CT: computed tomography; ED: emergency department.

## Competing interests

The authors declare that they have no competing interests.

## Authors’ contributions

The study was conceived and designed by CB, HH, SL, OB, SJ, JG, DR, JW, AS, JH-B and PE. OB and SL provided the statistical advice and OB, CB, HH and SL analysed the data. CB drafted the manuscript and HH, SL, OB, SJ, JG, DR, JW, AS, JH-B and PE contributed substantially to its critical revision for important intellectual content. CB takes overall responsibility for the paper. CB, HH, SL, OB, SJ, JG, DR, JW, AS, JH-B and PE have all approved the final version of the manuscript for publication.

## References

[B1] Demirhan R Onan B Oz K Halezeroglu S Comprehensive analysis of 4205 patients with chest trauma: a 10-year experience Interact Cardiovasc Thorac Surg 2009 9 450 453 10.1510/icvts.2009.206599 19541693

[B2] Battle CE Hutchings H Evans PA Risk factors that predict mortality in patients with blunt chest wall trauma: A systematic review and meta-analysis Injury 2012 43 8 17 10.1016/j.injury.2011.01.004 21256488

[B3] Ahmad MA Sante ED Giannoudis PV Assessment of severity of chest trauma: is there an ideal scoring system? Injury 2010 41 981 983 10.1016/j.injury.2010.08.004 20728883

[B4] Blecher GE Mitra B Cameron PA Fitzgerald M Failed emergency department disposition to the ward of patients with thoracic injury Injury 2008 39 586 591 10.1016/j.injury.2007.10.021 18336817

[B5] Alexander JQ Gutierrez CJ Mariano MC Vander Laan T Gaspard DJ Carpenter CL Stain SC Blunt chest trauma in the elderly patient: How cardiopulmonary disease affects outcome Am Surg 2000 66 854 857 10993616

[B6] Simon BJ Chu Q Emhoff TA Delayed haemothorax after blunt thoracic trauma: an uncommon entity with significant morbidity J Trauma 1998 45 673 676 10.1097/00005373-199810000-00005 9783603

[B7] Dubinsky I Low A Non-life threatening blunt chest trauma: appropriate investigation and treatment Am J Emerg Med 1997 15 240 243 10.1016/S0735-6757(97)90004-8 9148976

[B8] Battle CE Hutchings H Evans PA Expert opinion of the risk factors for morbidity and mortality in blunt chest wall trauma: Results of a national postal survey of Emergency Departments in the United Kingdom Injury 2013 44 56 59 10.1016/j.injury.2011.12.012 22227106

[B9] Moons KGM Altman DG Vergouwe Y Royston R Prognosis and prognostic research: application and impact of prognostic models in clinical practice BMJ 2009 338 1487 1490 10.1136/bmj.b606 19502216

[B10] Moons KGM Royston P Vergouwe Y Grobbee DE Altman DG Research methods and reporting. Prognosis and prognostic research: what, why and how? BMJ 2009 338 1317 1320 10.1136/bmj.b1317 19237405

[B11] Royston P Moons KGM Altman DG Vergouwe Y Research methods and reporting, Prognosis and prognostic research: developing a prognostic model BMJ 2009 338 1373 1377 10.1136/bmj.b1373 19336487

[B12] Altman DG Vergouwe Y Royston P Moons KGM Prognosis and prognostic research: validating a prognostic model BMJ 2009 338 1432 1435 10.1136/bmj.b1432 19477892

[B13] Peduzzi P Concato J Feinstein AR Holford TR The importance of events per independent variable in proportional hazards regression analysis J Clin Epidemiol 1995 48 1503 1510 10.1016/0895-4356(95)00048-8 8543964

[B14] Flagel BT Luchette FA Reed RL Esposito TJ Davis KA Santaniello JM Gamelli RL Half-a-dozen rib fractures: the breakpoint for mortality Surgery 2005 138 717 725 10.1016/j.surg.2005.07.022 16269301

[B15] Hoff SJ Shotts SD Eddy VA Morris JA Outcome of isolated pulmonary contusion in blunt trauma patients Am Surg 1994 60 138 142 8304646

[B16] Battle CE Hutchings H James K Evans PA The risk factors for the development of complications during the recovery phase following blunt chest wall trauma: a retrospective study Injury 2013 44 1171 1176 10.1016/j.injury.2012.05.019 22695321

[B17] Bouwmeester W Zuithoff NPA Mallett S Geerlings MI Vergouwe Y Steyerberg EW Altman DG Moons KG Reporting and Methods in Clinical Prediction Research: A Systematic Review PLoS Med 9 e1001221 10.1371/journal.pmed.1001221 PMC3358324 22629234

[B18] Vergouwe Y Steyerberg EW Eijkemans MJC Habbema DF Substantial effective sample sizes were required for external validation studies of predictive logistic regression models J Clin Epidemiol 2005 58 475 483 10.1016/j.jclinepi.2004.06.017 15845334

[B19] Janssen KJM Moons KGM Kalkman CJ Grobbeea DE Vergouwee Y Updating methods improved the performance of a clinical prediction model in new patients J Clin Epidemiol 2008 61 76 86 10.1016/j.jclinepi.2007.04.018 18083464

[B20] Lee SJ Lindquist K Segal MR Covinsky KE Development and validation of a prognostic index for 4-year mortality in older adults JAMA 2006 295 801 808 10.1001/jama.295.7.801 16478903

[B21] Wutzler S Wafaisade A Maegele M Laurer H Geiger EV Walcher F Barker J Lefering R Marzi I Lung organ failure score: probability of severe pulmonary organ failure after multiple injuries including chest trauma Injury 2012 43 1507 1512 10.1016/j.injury.2010.12.029 21256489

[B22] Toll DB Janssen KJM Vergouwe Y Moons KGM Validation, updating and impact of clinical prediction rules: a review J Clin Epidemiol 2008 61 1085 1094 10.1016/j.jclinepi.2008.04.008 19208371

[B23] Steyerberg EW Borsboom GJ Houwelingen HC Eijkemans MJ Habbema JD Validation and updating of predictive logistic regression models: a study on sample size and shrinkage Statist Med 2004 23 2567 2586 10.1002/sim.1844 15287085

[B24] Bergeron E Lavoie A Clas D Moore L Ratte S Tetreault S Lemaire J Martin M Elderly trauma patients with rib fractures are at greater risk of death and pneumonia J Trauma 2003 54 478 485 10.1097/01.TA.0000037095.83469.4C 12634526

[B25] Easter A Management of patients with multiple rib fractures Am J Crit Care 2001 10 320 327 11548565

[B26] Davis S Affatato A Blunt chest trauma: utility of radiological evaluation and effect on treatment patterns Am J Em Med 2006 24 482 486 10.1016/j.ajem.2006.03.022 16787809

